# Research progress on predictive models for malnutrition in cancer patients

**DOI:** 10.3389/fnut.2024.1438941

**Published:** 2024-08-21

**Authors:** Pengcheng Zheng, Bo Wang, Yan Luo, Ran Duan, Tong Feng

**Affiliations:** ^1^Clinical Medical College, Chengdu Medical College, Chengdu, China; ^2^Department of Respiratory and Critical Care Medicine, The First Affiliated Hospital of Chengdu Medical College, Chengdu, China; ^3^Key Laboratory of Geriatic Respiratory Diseases of Sichuan Higher Education Institute, Chengdu, China; ^4^Department of Oncology, The First Affiliated Hospital of Chengdu Medical College, Chengdu, China; ^5^The Second School of Clinical Medicine, Southern Medical University, Guangzhou, China

**Keywords:** cancer patients, nutrition status, prediction models, machine learning, nomogram

## Abstract

Disease-related malnutrition is a prevalent issue among cancer patients, affecting approximately 40–80% of those undergoing treatment. This condition is associated with numerous adverse outcomes, including extended hospitalization, increased morbidity and mortality, delayed wound healing, compromised muscle function and reduced overall quality of life. Moreover, malnutrition significantly impedes patients’ tolerance of various cancer therapies, such as surgery, chemotherapy, and radiotherapy, resulting in increased adverse effects, treatment delays, postoperative complications, and higher referral rates. At present, numerous countries and regions have developed objective assessment models to predict the risk of malnutrition in cancer patients. As advanced technologies like artificial intelligence emerge, new modeling techniques offer potential advantages in accuracy over traditional methods. This article aims to provide an exhaustive overview of recently developed models for predicting malnutrition risk in cancer patients, offering valuable guidance for healthcare professionals during clinical decision-making and serving as a reference for the development of more efficient risk prediction models in the future.

## 1 Introduction

Cancer patients frequently encounter malnutrition, predominantly as a result of the cancer itself, associated pain, symptoms, adverse lifestyle choices, and the side effects arising from treatment (1). Nutritional support for cancer patients has emerged as a pivotal focus and an integral part of frontline therapy. Recent findings indicate that early nutritional and psychological interventions can reduce the mortality risk in patients with advanced esophageal and gastric cancers by up to 32% (2). Hence, screening for malnutrition risk has become a critical aspect of nutritional management, and the selection of effective and suitable predictive tools is essential for evaluating the nutritional status of cancer patients. This article provides a comprehensive review of research on predictive models for malnutrition in cancer patients, both domestically and internationally, with the aim of offering a reference for the establishment of relevant predictive models.

## 2 Predictive factors

Currently, extensive research has been conducted on the risk factors for malnutrition in cancer patients both domestically and internationally. These studies have provided valuable insights for identifying predictive factors. Demographic variables such as age are closely associated with malnutrition in cancer patients. Several studies have indicated that individuals over the age of 70 are prone to malnutrition, potentially due to the compromised basic health and cardiopulmonary function in elderly patients. Moreover, the weakened immune system in elderly patients makes it difficult for them to combat the systemic inflammatory response induced by cancer.

Regarding the relationship between weight changes and prognosis in cancer patients, Martin et al. (3) developed a cancer grading system incorporating two dimensions: Weight Loss (WL) percentage and Body Mass Index (BMI), linking it to survival time. The researchers employed a 5 × 5 matrix analysis to outline 25 possible combinations of WL percentage and BMI to predict survival rates at five different levels. This BMI-adjusted weight loss grading system is a useful tool for predicting survival rates as it is independent of tumor site, stage, or clinical presentation, solely reflecting differences in patient survival rates.

Certain inflammatory markers, such as C-reactive protein and neutrophils, have been shown to be associated with malnutrition in cancer patients. The Neutrophil to Lymphocyte ratio (NLR) is an indicator of the level of inflammation in the body. On one hand, a more intense inflammatory response in cancer patients leads to greater nutrient consumption, which can exacerbate stress trauma, such as surgery-related trauma, increasing the chance of postoperative infection and promoting the occurrence of postoperative malnutrition (4). On the other hand, the presence of neutrophils is associated with tumor growth and metastasis because neutrophils can produce soluble cytokines, various proteases, and inhibit the functions of effector T cells and NK cells. The reduction in lymphocyte count signifies decreased immune function and surveillance capability, making tumors more prone to metastasis (5). Accelerated tumor growth or metastasis directly exacerbates nutrient consumption.

Prealbumin (PAB), synthesized by liver cells, is more sensitive to malnutrition compared to albumin and transferrin due to its half-life of only 12 h. Research by Aoyama et al. suggests that prealbumin can serve as a representative indicator for evaluating the postoperative nutritional status of cancer patients and is related to recurrence and survival rates (6), while Zu et al. (7) confirmed that prealbumin levels at admission are an independent risk factor for long-term prognosis in cancer patients.

Phase angle (PA) is a parameter obtained through bioelectrical impedance analysis (BIA). Previous studies have shown that PA is a reliable indicator for assessing nutritional status and a valuable prognostic biomarker in cancer (8). In patients with head and neck cancer, it was observed that PA values decrease before weight loss or changes in BMI occur (9).

Cardiac function classification is based on cardiac color Doppler parameters, dividing cardiac function into three levels according to left ventricular ejection fraction and the degree of diastolic dysfunction. Kinugawa and Fukushima (10) believe that chronic heart failure patients often experience malnutrition due to changes in systemic metabolism and increased body consumption, with an incidence rate of 16–62%. Patients undergoing cancer surgery are more likely to experience insufficient intake, loss of appetite, and the risk of postoperative malnutrition because, in the presence of concurrent heart failure, patients’ intake and exercise tolerance decrease. Sze et al. (11) found that chronic heart failure exacerbates gastrointestinal congestion and intestinal edema symptoms in cancer patients, affecting nutrient absorption and increasing the occurrence of malnutrition. However, whether it can be used as an early predictive factor for malnutrition and included in predictive models requires further validation.

## 3 Predictive models

### 3.1 Statistical models

The logistic regression model is utilized to analyze the impact of independent variables on a binary dependent variable. By inputting a linear combination of the independent variables into a logistic function, it converts the result into a probability to predict the likelihood of a binary outcome. This model is widely used for the analysis, prediction, and classification of disease risk factors.

Dai et al. (12) retrospectively collected clinical data from 344 gastric cancer patients who underwent laparoscopic surgery, dividing the data into training and validation sets in a 7:3 ratio. Using logistic regression, the researchers developed a nutritional risk assessment model for gastric cancer patients post-gastrectomy. The model incorporated factors such as tumor lymph node metastasis staging, cardiac function classification, prealbumin levels, neutrophil to lymphocyte ratio, and enteral nutrition within 48 h post-surgery. The study results demonstrated that the model’s C-index was 0.84 (95% CI, 0.79–0.89), and the area under the receiver operating characteristic curve (AUC) was 0.840 for the training set and 0.854 for the validation set, indicating superior performance compared to the NRS2002 Nutritional Risk Screening tool (NRS2002). The calibration curve Brier scores were 0.159 and 0.195, and the Hosmer-Lemeshow test chi-square values were 14.070 and 1.989 (*p* > 0.05), signifying good model fit. Decision Curve Analysis (DCA) of the training set model indicated good clinical applicability, showing that within the 10–85% threshold probability range, the model outperformed NRS2002.

Yin et al. (13) through a multicenter, observational cohort study, performed a comparative analysis of data from 1,219 lung cancer patients. They employed a traditional logistic regression method to construct a predictive model incorporating six variables: gender, body mass index, weight loss within 6 months, weight loss after 6 months, calf circumference, and the ratio of handgrip strength to body weight. The model demonstrated an AUC value of 0.982 (95% confidence interval, 0.969–0.995), with similarly outstanding performance in the validation cohort. The indicators used in these models are non-invasive and cost-effective, with data easily obtainable through simple surveys and basic measurements taken at patient admission.

Tang et al. (14) included 506 outpatient colorectal cancer patients, collecting data on demographics, anthropometric measurements, laboratory results, patient-reported symptoms, cancer history, socioeconomic status, and comorbidities. They identified predictive factors for malnutrition using a logistic regression model (14). Significant predictive factors for malnutrition included age, body mass index, Eastern Cooperative Oncology Group (ECOG) performance status score, metastatic disease, albumin levels <3.0 g/dL, fatigue, and changes in stool/bowel habits. As the malnutrition risk score increased (from 0 points to 9–10 points), the risk of malnutrition rose from 11 to 100%. The model demonstrated an AUC value of 0.745 (95% CI, 0.697–0.793).

Yu et al. (15) collected computed tomography (CT) scan data from 120 cervical cancer patients before they underwent chemoradiotherapy. By analyzing non-enhanced CT images, they extracted radiological features of the L3 psoas major muscle. The research team utilized the least absolute shrinkage and selection operator (LASSO) to predict malnutrition in the training dataset, identifying optimal features and constructing a radiomics score (rad-score) formula. In the clinical model, researchers used a binary logistic regression model to analyze key clinical factors, combining radiological features with clinical risk factors to develop a radiomics-based nomogram. Multivariate analysis revealed that, in addition to the rad-score, age and ECOG performance status were independent predictors of malnutrition. In the combined model, the AUC for the training set and validation set increased to 0.972 and 0.805, respectively. Decision Curve Analysis (DCA) also confirmed the clinical utility of the combined model. Given the retrospective design and small sample size of this study, future research will require large-sample prospective external validation.

Regarding sample size, it is recommended that the EPV (Events Per Variable) be above 20 as the minimum sample size for model development. A study with an EPV less than 10 is considered inadequate (15). Insufficient EPV can lead to a high risk of overfitting and prediction bias. This means that although the reported AUC is close to 1, the performance of these models on a new dataset could be significantly worse. Future research should determine an appropriate sample size, as different predictive modeling studies and different modeling techniques require different EPV values. For example, the EPV for model validation studies should be above 100.

The handling of missing data is a prevalent and increasingly significant issue in medical science research. Simply excluding participants with missing data from the analysis, referred to as complete case analysis, can introduce biases in the predictor-outcome relationships and model performance. The mentioned study did not report any information on missing data. In such cases, participants with missing data are more likely to be excluded from statistical analysis because statistical software tends to automatically omit individuals with any missing values. Multiple imputation can serve as one of the solutions for addressing missing data. The main advantage of multiple imputation is that it yields accurate standard errors and *p*-values, making it considered the most appropriate method for handling missing data.

A study dichotomized continuous predictors (14). While dichotomizing continuous predictors can enhance clinical interpretability and maintain simplicity, it is a suboptimal choice due to information loss, reduced predictive capability, and the potential for overestimating model performance. It is recommended to retain predictors as continuous variables and to examine the linear relationship between predictors and outcomes (e.g., using restricted cubic splines or fractional polynomials). If researchers consider categorization in their study, they should divide continuous predictors into four or more groups based on widely accepted cut-off points.

After developing or validating a predictive model, testing its performance is a critical step. Different measures can be employed to evaluate model performance, and it is recommended that all predictive model papers report calibration and ROC curves. However, only two studies reported both calibration and ROC curves (12, 13). For calibration, calibration curves are more suitable than statistical tests (such as the Hosmer-Lemeshow test), as the Hosmer-Lemeshow test cannot indicate the direction or magnitude of calibration bias. For modeling studies, both external and internal validation are essential. Four studies randomly split their datasets into training and validation groups (12, 13, 15, 19). However, this approach is particularly suboptimal with small sample sizes, as it merely creates two smaller but similar datasets by chance and does not utilize all available data to develop the predictive model. It is recommended to use bootstrapping and cross-validation techniques for internal validation to correct for the optimistic bias of predictive models. To ensure the generalizability of predictive models, external validation is required.

### 3.2 Machine learning models

With the continuous advancement of computer technology and the enhancement of clinical big data, the application of machine learning in disease diagnosis, prognosis evaluation, and image recognition has become increasingly prevalent (16). Machine learning algorithms have become a research hotspot in data analysis due to their ability to automatically identify complex relationships between features, facilitate predictive analysis, and effectively leverage multidimensional data from electronic medical records systems (17). Consequently, researchers have started employing machine learning models to predict malnutrition in cancer patients (see Table 1).

**Table 1:** Prediction models for malnutrition risk in cancer patients.

Researchers	Year(s)	Sample size	Model validation methods	Predictive factors	Modeling methods and AUC statistics
Dai (12)	2023	Training Set:242 Validation Set:102	Internal Validation	Tumor Lymph Node Metastasis Stage, Cardiac Function Classification, Prealbumin, Neutrophil to Lymphocyte Ratio, Enteral Nutrition within 48 Hours Post-Surgery	Logistic Regression AUC: 0.840
Yu (15)	2023	Training Set:84 Validation Set:36	Internal Validation	Rad Score, Age, ECOG Score	Logistic Regression AUC:0.972
Tang (14)	2021	Training Set:504	None	Age, BMI, ECOG Metastatic Disease, Albumin <3.0 g/dL, Fatigue, and Changes in Stool/Bowel Habits	Logistic Regression AUC:0.745
Yin (13)	2021	Training Set:914 Validation Set:305	Internal Validation	Gender, BMI, Weight Loss within 6 Months, Weight Loss after 6 Months, Calf Circumference, and the Ratio of Handgrip Strength to Body Weight	Logistic Regression AUC:0.982
Yin (19)	2022	Training Set:2998 Validation Set:1000	Internal Validation	Age, Weight Loss within 6 Months, BMI, Calf Circumference, and NRS 2002	Decision Tree AUC:0.964
Zhang (18)	2022	Training Set:702	None	Age, Tumor Type, BMI, and Left Arm PA	Decision Tree AUC:0.813

AUC refers to the Area Under the Receiver Operating Characteristic Curve, BMI refers to Body Mass Index, PA refers to Phase Angle, NRS 2002 refers to Nutritional Risk Screening 2002, and ECOG refers to Eastern Cooperative Oncology Group Performance Status.

Zhang et al. (18) conducted a retrospective analysis of medical records from 702 cancer patients, selecting age, tumor type, left arm phase angle, and BMI as predictive factors to construct decision tree and random forest artificial neural network models. The results demonstrated that the model exhibited good performance, with an AUC of 0.813, a sensitivity of 75.9%, and a specificity of 73.3%. The actual and predicted survival curves were largely consistent. However, due to the limitations of the retrospective study, the researchers were unable to collect information on smoking, alcohol consumption, patient education level, and income, which could potentially enhance the model’s predictive capability. Additionally, as this was a single-center study, multicenter studies are required to validate the predictive model.

Yin et al. (19) collected data from 3,998 cancer patients across multiple centers and employed a decision tree algorithm to construct a model incorporating five key predictive factors: age, weight loss within 6 months, body mass index, calf circumference, and NRS 2002. The model exhibited excellent discriminative ability, with an AUC of 0.964. Subgroup analysis indicated that the model had significant advantages across various tumor types. However, the model was also constrained by the retrospective study design, as it did not include clinical indicators of inflammatory status, such as red cell distribution width or long-term steroid use, which could potentially influence the number of patients ultimately diagnosed with malnutrition.

Current studies on machine learning modeling processes often lack completeness, typically missing detailed tuning procedures and model interpretation, which impedes understanding of the models. In the medical domain, the importance of interpretability is especially significant as it directly impacts patient health and safety. If the decision-making processes of medical predictive models are transparent and interpretable, both doctors and patients are more likely to trust the recommendations made by these models. This trust is essential for the acceptance and practical application of the models. By elucidating the predictive outcomes of the models, doctors can better grasp the reasoning behind certain treatment options, leading to more precise clinical decisions. This comprehensive understanding allows doctors to integrate their professional knowledge with the specific conditions of patients, facilitating personalized treatment. Medical data may harbor biases related to race, gender, or age, which unchecked models might amplify. Interpretable models aid in identifying and mitigating these potential biases, ensuring equitable treatment for all patients.

## 4 The risk of bias in predictive models

Table 2 employs the Prediction model risk of bias assessment tool (PROBAST) to evaluate the risk of bias and applicability concerns for each model. Across all models, the assessments related to study participants and predictors showed low risk of bias and applicability concerns. However, many predictive models demonstrated high or unclear risk of bias in the outcome and analysis domains. Upon comprehensive evaluation, it was found that six models had a high risk of bias. Furthermore, most studies failed to fully describe the completeness of the participant data, the information regarding missing data, and the statistical methods used to address missing data. One model used univariate analysis to identify predictors during the selection process. Including only statistically significant variables from univariate analysis as predictors can result in the omission of important risk factors, thereby increasing the risk of bias (20).

**Table 2:** Assessment results of prediction model bias risk assessment tools.

Model	Risk of bias				Applicability risk			Overall	
	Research object	Predictive factors	result	analysis	Research object	Predictive factors	result	Risk of bias	Applicability risk
Dai (12)	−	−	+	+	−	−	−	+	−
Yu (13)	−	−	?	+	−	−	?	+	?
Tang (14)	−	−	+	+	−	−	−	+	−
Yu (15)	−	−	?	+	−	−	?	+	?
Yin (19)	−	−	+	+	−	−	−	+	−
Zhang (18)	−	−	+	+	−	−	−	+	−

“–” indicates low risk, “+” indicates high risk, “?” indicates unclear.

## 5 Summary

Currently, there is a notable absence of multicenter, large-sample cohort validated malnutrition predictive models for cancer patients. With the ongoing advancements in medical information technology and artificial intelligence, machine learning has shown superiority in data processing over traditional modeling methods. Future research could explore dynamic risk assessment predictive models by collecting data from patients at multiple time points, allowing for continuous risk assessment, timely detection of changes in condition, and early intervention to prevent deterioration. Simultaneously, there is a need to pursue interpretable machine learning models, using artificial intelligence to develop advanced interpretable predictive models that provide healthcare professionals with insights into risk predictions. Efforts should continue to bolster the construction of large clinical databases, integrating big data with computer technology to continuously refine high-quality clinical predictive models. This will facilitate the early detection of malnutrition in cancer patients, reduce patient suffering and medical costs, and ultimately improve patient prognosis.

## Author contributions

PZ: Writing – original draft. BW: Writing – original draft. YL: Writing – original draft. RD: Writing – review & editing. TF: Writing – review & editing.
